# Overweight and obesity among adults in Germany – Results from GEDA 2019/2020-EHIS

**DOI:** 10.25646/10293

**Published:** 2022-09-14

**Authors:** Anja Schienkiewitz, Ronny Kuhnert, Miriam Blume, Gert B.M. Mensink

**Affiliations:** Robert Koch Institute, Berlin Department of Epidemiology and Health Monitoring

**Keywords:** OVERWEIGHT, OBESITY, SELF-REPORTING, HEALTH MONITORING, GEDA 2019/2020-EHIS

## Abstract

**Background:**

Overweight and obesity and their associated secondary diseases are of high public health relevance.

**Methods:**

Self-reported body weight and body height data are available in the study German Health Update (GEDA 2019/2020-EHIS). The body mass index (BMI, kg/m^2^) was calculated and overweight (including obesity, BMI ≥25 kg/m^2^) and obesity (BMI ≥30 kg/m^2^) were derived.

**Results:**

According to this self-report, 53.5% of adults in Germany are overweight, men more often than women. The obesity prevalence for both sexes is 19.0%. The prevalence of overweight and obesity increases with age in both women and men. Obesity is significantly more prevalent in low education groups compared to high education groups. Compared to GEDA 2012, the prevalence of overweight is unchanged, but the obesity prevalence has continued to increase, particularly among 45- to 64-year-olds.

**Conclusion:**

The prevention potential of avoiding overweight and obesity remains high.

## Introduction

Overweight is a body weight which exceeds the normal body weight for a given body height. Severe overweight is also known as obesity and is classified as a disease by the World Health Organization (WHO) [1]. Obesity is a risk factor for many secondary diseases such as type 2 diabetes, cardiovascular diseases, several types of cancer, musculoskeletal disorders and is associated with a higher risk of premature death [2, 3]. The risk of other diseases increases with increasing severity of obesity. Obesity and its secondary diseases are a significant public health problem, both nationally and internationally, and a major challenge for the health care system: OECD countries spend approximately 8.4% of their health care expenditures on the treatment of obesity-related diseases [4]. Worldwide, the proportion of people with obesity has tripled since 1975 [5], and in Germany the prevalence has also been increasing continuously since 1990 [6, 7]. The extent to which this increase in the prevalence of overweight and obesity in the population will continue – also against the background of the COVID-19 pandemic – can be assessed using current interview data from the GEDA 2019/2020-EHIS study. This article presents current overweight and obesity prevalence by sex, age, and education groups.

**GEDA 2019/2020-EHIS**

Fifth follow-up survey of the German Health Update

**Data holder:** Robert Koch Institute

**Objectives:** Provision of reliable information on the health status, health behaviour and health care of the population living in Germany, with the possibility of European comparisons

**Study design**: Cross-sectional telephone survey

**Population:** German-speaking population aged 15 and older living in private households that can be reached via landline or mobile phone

**Sampling:** Random sample of landline and mobile telephone numbers (dual-frame method) from the ADM sampling system (Arbeitskreis Deutscher Markt- und Sozialforschungsinstitute e.V.)

**Sample size:** 23,001 respondents

**Study period:** April 2019 to September 2020

**GEDA survey waves:**

▶ GEDA 2009
▶ GEDA 2010
▶ GEDA 2012
▶ GEDA 2014/2015-EHIS
▶ GEDA 2019/2020-EHIS

Further information in German is available at www.geda-studie.de

## Indicator

The German Health Update (GEDA) is a nationwide cross-sectional survey of the resident population living in Germany. The fifth follow-up survey, GEDA 2019/2020-EHIS, took place between April 2019 and September 2020. In GEDA 2019/2020-EHIS, 23,001 participants aged 15 years and older were interviewed about their health status, health care, health behaviour, and their demographic and socioeconomic background. Interview duration was approximately 40 minutes. The response rate was 21.6%. A detailed description of the GEDA 2019/2020-EHIS methodology can be found in the article German Health Update (GEDA 2019/2020-EHIS) – Background and methodology in issue 3/2021 of the Journal of Health Monitoring [8].

In GEDA 2019/2020-EHIS, body height and body weight were asked in a telephone interview using a programmed, fully structured questionnaire (Computer Assisted Telephone Interview, CATI). The question on body height was: ‘How tall are you when you are not wearing shoes?’ The information was given in cm. The body weight question was: ‘How much do you weigh when you are not wearing clothes and shoes? Please indicate your body weight in kg. Pregnant women, please indicate your weight before pregnancy.’

In GEDA 2019/2020-EHIS, the data on body weight and body height are self-reported, and in this situation body weight is often underestimated compared to standardised measured values, while body height tends to be overestimated. As a result, the body mass index (BMI; see Info box) calculated using self-reported data is lower than a BMI calculated using measured body weight data [9], so that overweight and obesity prevalence from the GEDA time series is lower than those from measured data from examination surveys. Therefore, the current prevalence of overweight (including obesity) and obesity may be underestimated.

Gender identity was used in GEDA 2019/2020-EHIS to describe gender differences [10]. Participants were able to indicate which sex they felt they belonged to. Among participants 18 years and older, 11,959 indicated a female identity and 10,687 indicated a male identity. 62 participants indicated a different gender identity or did not provide any information. In the analyses stratified by sex, individuals with a different gender identity or no indication are not considered. The present analyses are based on data from 22,414 participating individuals aged 18 years and older with valid information on body weight and height; 11,736 women and 10,618 men are used for analyses by sex, age education groups.

To correct for deviations of the sample from the population structure, the analyses were performed applying a weighting factor. First, a design weighting was performed for the different selection probabilities (mobile and fixed network) and then an adjustment was made to the official population figures in terms of age, sex, federal state and district type (as of: 31/12/2019). In addition, the distribution of education was adjusted to the distribution in the German Microcensus (2017) according to the International Standard Classification of Education (ISCED classification) [11].

In this article, prevalence with 95% confidence intervals (95% CI) for underweight, normal weight, overweight without obesity, obesity and overweight (including obesity) are reported in tabular form. Due to space limitations, in the text, only obesity and overweight (including obesity) are described by sex, age and education groups. When comparing current prevalence from GEDA 2019/2020-EHIS with previous GEDA surveys, it must be considered that not only the sampling method (population registry sample, random sample of mobile and landline phone numbers) and survey mode (paper, telephone, online) but also the wordings of some questions have changed slightly. For this reason, a comparison with GEDA 2014/2015-EHIS is limited [12] and the survey GEDA 2012 is used for comparing trends in prevalence over time. To evaluate trends, age-standardised prevalence rates were calculated at both survey time points, and the difference was tested using univariable logistic regression. Even though weighting is used to adjust the study population to the population composition at the time of the survey according to the above criteria, deviations in the study population with regard to other characteristics cannot be excluded. Due to space limitations in this fact sheet, comparisons between GEDA 2012 and GEDA 2019/2020-EHIS are made only for the overall prevalence and prevalence rates stratified by sex for the indicators overweight (including obesity) and obesity. A significant difference is assumed if the p-value, taking into account the weighting and the survey design, is less than 0.05.

**Info box Body Mass Index (BMI)**

To calculate the BMI, the body weight of a person (in kilograms) is divided by the square of the body height (measured in meters). BMI categories are defined according to World Health Organization (WHO) classification [1] as follows:

Underweight BMI<18.5 kg/m^2^

Normalweight: 18.5–<25 kg/m^2^

Overweight (including obesity): BMI≥25.0 kg/m^2^

Obesity: BMI≥30.0 kg/m^2^,

Obesity Class 1: BMI 30–<35 kg/m^2^,

Obesity Class 2: BMI 35–<40 kg/m^2^,

Obesity Class 3: BMI≥40.0 kg/m^2^.

## Results and classification

In Germany, 53.5% of the population (46.6% of women and 60.5% of men) is overweight (including obesity). Obesity is present in 19.0% of adults. While significantly more men than women are overweight (including obese), there is no difference in obesity prevalence between the sexes (Table 1).

It can be seen that the age-standardised prevalence of overweight (including obesity) has remained high in recent years. In 2012 it was 53.0% (women 46.3%, men 59.7%). Compared to GEDA 2012, there is no significant change. In contrast, obesity prevalence has continued to increase since GEDA 2012 for both women (+2.5 percentage points) and men (+2.1 percentage points). Accordingly, currently nearly 13 million adults in Germany are obese.

Prevalence rates of overweight (including obesity) and obesity are similar as observed in official statistics, for which data is also based on self-reports by participants. According to the German Microcensus (2017), 52.7% of adults were overweight (including obese), and 16.3% were obese [13]. Compared to the first survey in 1999, there was an increase of 3.5 percentage points for women and 6 percentage points for men. Thus, the GEDA 2019/2020-EHIS results confirm the upward trends in obesity prevalence observed with other population-based data.

According to GEDA 2019/2020-EHIS, 13.2% (12.5%–13.9%) of adults have obesity Class 1, 4.0% (3.6%–4.5%) have obesity Class 2, and 1.8% (1.5%–2.2%) have obesity Class 3 (for definitions see Info box). Compared to GEDA 2012, there has been an increase of 1.1 percentage points for Class 1, 0.7 percentage points for Class 2, and 0.5 percentage points for Class 3. Nationwide ambulatory claims data also show that the prevalence of diagnosed obesity increased significantly between 2009 and 2018: from 3.5% to 13.6% for Class 1, from 1.7% to 11.7% for Class 2, and from 1.1% to 7.1% for Class 3 [14]. However, the significant prevalence increase in ambulatory claims data between 2009 and 2018 is also due to coding: the proportion of non-specific coded obesity diagnoses decreased over time, and coding is increasingly differentiated by obesity categories (Class 1–3). This is another reason why the further spread of obesity of all class levels can be observed over the past decade.

The prevalence of overweight (including obesity) increases with age, and the proportion of women and men affected by obesity also increases steadily over the life course: While only about 10% of women and men in the 18- to 29-year-old age group are affected by obesity, this proportion rises to more than 20% among 45- to 64-year-olds. These age- and gender-specific trends are also consistent with results from earlier surveys conducted by the Robert Koch Institute [7]. A statistically significant increase in obesity prevalence has been observed since GEDA 2012, particularly among young women in the 18- to 29-year-old age group (from 5.7% to 10.0%) and in the 45- to 64-year-old age group: from 19.3% to 22.9% among women and from 20.4% to 23.8% among men. Since GEDA 2012, the prevalence of obesity in the age group 65 years and older is unchanged. While in recent years there has been an increase primarily in young adulthood, there is now also an increase in the middle age group.

Obesity is significantly more common in the low education group compared to the high education group, a fact that has already been described in previous population-based surveys internationally [15, 16] and also for Germany [17]: More than twice as many women and men in the low education group compared to the high education group are affected by obesity, with the exception of men in the age group 65 years and older. Also, for the indicator overweight (including obesity), there is a 1.5 times higher prevalence among women in the low education group compared to women in the high education group, again with the exception of those 65 years and older. Among men, these differences are observed only among those aged 45 to 64 years. The extent to which these differences have changed since GEDA 2012 will be revealed by further analysis of the GEDA data. However, results from the period 1990 to 2011 indicate an increase in health inequalities due to the differential increase in obesity prevalence across socioeconomic status groups [17].

Thus, it can be stated that the goal of the German Sustainable Development Strategy from 2016 [18], to prevent the proportion of the adult population with obesity from increasing further, has not yet been achieved. Comparison of GEDA 2012 and GEDA 2019/2020-EHIS shows a further increase. Whether and how substantial the months with strict containment measures due to the COVID-19 pandemic had an impact on the prevalence of overweight and obesity, cannot be determined from these survey data. Initial analysis from GEDA 2019/2020-EHIS on body weight and BMI show that there was an increase in body weight and BMI between April to August 2020 compared to April to August 2019 [19], but this increase did not continue between October 2020 and January 2021. Nevertheless, compared with the pre-pandemic period, mean BMI increased by 0.3 units and body weight increased by 0.8 kg [20]. This weight gain appears small at first, but it is substantially higher than the average mean weight gain per year for the 45- to 64-year-old cohort participants: It is only 250 g for men and 240 g for women [21].

Future structured treatment programs for those affected by obesity (so-called Disease Management Programmes, DMPs) should help to improve obesity treatment. A DMP for obesity is currently being developed. The huge prevention potential of overweight and obesity should be emphasised and prevention measures aimed at changing individual health behaviour as well as setting based measures to reduce social health inequalities should be addressed.

## Key statement

- According to self-reported data, 46.6% of women and 60.5% of men in Germany are overweight (including obesity), and 19.0% of adults are affected by obesity.
- With increasing age, the prevalence of overweight (including obesity) and obesity increases in both women and men.
- Obesity is significantly more prevalent in low education groups compared with high education groups.
- Compared to GEDA 2012, obesity prevalence has further increased. A significant increase is particularly evident among adults aged 45 to 64.

## Figures and Tables

**Table 1:** Underweight, normal weight, overweight and obesity according to sex, age group and education group (n=11.736 women, n=10.618 men) Source: GEDA 2019/2020-EHIS

	Underweight		Normal weight		Overweight (without obesity)		Obesity		Overweight (including obesity)	
	BMI	<18.5 kg/m^2^	18.5-<25.0 kg/m^2^		25.0-<30 kg/m^2^		≥30 kg/m^2^		≥25 kg/m^2^	
	%	(95% CI)	%	(95% CI)	%	(95% CI)	%	(95% CI)	%	(95% CI)
Total	2.3	(2.0–2.7)	44.2	(43.2–45.2)	34.5	(33.5–35.4)	19.0	(18.2–19.8)	53.5	(52.5–54.5)
**Women**	**3.4**	**(2.9–4.0)**	**50.0**	**(48.6–51.4)**	**27.6**	**(26.5–28.9)**	**19.0**	**(17.9–20.1)**	**46.6**	**(45.3–48.0)**
**18–29 years**	7.2	(5.2–9.9)	66.6	(62.4–70.6)	16.2	(13.3–19.6)	10.0	(7.5–13.2)	26.2	(22.5–30.2)
Low education group	15.5	(8.5–26.6)	56.9	(45.6–67.6)	19.9	(12.5–30.2)	7.7	(3.6–15.7)	27.6	(18.9–38.4)
Medium education group	4.4	(2.7–7.2)	66.7	(61.1–71.8)	16.1	(12.5–20.7)	12.7	(9.2–17.4)	28.9	(24.0–34.4)
High education group	8.4	(5.2–13.1)	75.4	(68.9–81.0)	13.1	(8.9–18.8)	3.1	(1.7–5.6)	16.2	(11.7–22.0)
**30–44 years**	4.1	(3.0–5.7)	55.1	(51.9–58.3)	26.2	(23.5–29.1)	14.6	(12.4–16.9)	40.8	(37.7–43.9)
Low education group	2.2	(0.4–10.3)	50.4	(37.3–63.4)	28.0	(17.7–41.3)	19.4	(11.3–31.3)	47.4	(34.6–60.6)
Medium education group	4.1	(2.4–6.8)	50.0	(45.7–54.4)	29.5	(25.8–33.5)	16.4	(13.6–19.7)	45.9	(41.6–50.2)
High education group	5.1	(3.6–7.2)	65.6	(62.1–69.0)	20.1	(17.4–23.1)	9.1	(7.2–11.5)	29.3	(26.1–32.7)
**45–64 years**	2.0	(1.5–2.6)	47.0	(44.9–49.0)	28.2	(26.4–30.1)	22.9	(21.1–24.8)	51.1	(49.0–53.1)
Low education group	1.2	(0.3–3.9)	36.3	(29.2–44.1)	23.5	(17.7–30.5)	39.0	(31.7–46.9)	62.5	(54.8–69.7)
Medium education group	1.8	(1.3–2.6)	47.0	(44.4–49.5)	29.8	(27.6–32.2)	21.4	(19.4–23.6)	51.2	(48.7–53.7)
High education group	2.9	(2.1–3.9)	54.6	(52.1–57.1)	27.0	(24.9–29.2)	15.5	(13.7–17.5)	42.5	(40.1–45.0)
**≥65 years**	2.6	(1.9–3.5)	41.1	(38.8–43.5)	34.0	(31.8–36.3)	22.4	(20.4–24.5)	56.3	(53.9–58.7)
Low education group	3.6	(1.9–6.6)	40.1	(34.7–45.8)	31.4	(26.4–36.8)	24.9	(20.4–30.1)	56.3	(50.6–61.8)
Medium education group	1.8	(1.3–2.5)	39.5	(37.0–42.1)	35.8	(33.3–38.3)	22.9	(20.7–25.3)	58.7	(56.1–61.2)
High education group	3.1	(2.2–4.4)	50.2	(47.3–53.1)	33.8	(31.1–36.6)	12.9	(11.1–14.9)	46.7	(43.8–49.5
**Men**	**1.3**	**(0.9–1.7)**	**38.3**	**(36.9–39.7)**	**41.3**	**(39.9–42.8)**	**19.1**	**(18.0–20.3)**	**60.5**	**(59.0–61.9)**
**18–29 years**	2.7	(1.8–4.1)	60.7	(56.9–64.4)	27.2	(23.9–30.7)	9.3	(7.2–12.1)	36.5	(32.9–40.3)
Low education group	5.6	(2.9–10.5)	56.7	(47.9–65.1)	21.7	(15.4–29.7)	16.0	(9.8–25.1)	37.7	(29.5–46.7)
Medium education group	2.2	(1.3–3.9)	61.0	(55.9–65.8)	28.9	(24.4–33.8)	7.9	(5.6–11.1)	36.8	(32.0–41.8)
High education group	0.8	(0.3–2.2)	65.0	(59.0–70.6)	28.3	(23.1–34.1)	5.9	(3.6–9.6)	34.2	(28.6–40.2)
**30–44 years**	1.4	(0.7–2.9)	40.4	(37.2–43.6)	39.5	(36.3–42.7)	18.7	(16.3–21.5)	58.2	(54.9–61.4)
Low education group	3.8	(0.9–14.1)	36.7	(25.2–49.9)	33.7	(22.7–46.7)	25.9	(16.4–38.3)	59.5	(46.4–71.5)
Medium education group	1.4	(0.6–3.5)	37.0	(32.5–41.8)	40.2	(35.6–45.0)	21.3	(17.7–25.4)	61.5	(56.8–66.1)
High education group	0.5	(0.1–1.6)	47.4	(43.7–51.1)	40.0	(36.4–43.6)	12.1	(10.1–14.5)	52.1	(48.4–55.8)
**45–64 years**	1.0	(0.5–1.9)	30.4	(28.3–32.5)	44.9	(42.6–47.2)	23.8	(21.7–26.0)	68.7	(66.5–70.8)
Low education group	2.4	(0.5–10.2)	16.9	(10.9–25.2)	44.1	(34.3–54.5)	36.6	(27.4–47.0)	80.8	(71.9–87.4)
Medium education group	1.2	(0.6–2.3)	30.5	(27.5–33.7)	43.2	(39.9–46.4)	25.2	(22.4–28.2)	68.3	(65.1–71.4)
High education group	0.2	(0.1–0.5)	34.5	(32.3–36.9)	48.3	(45.9–50.7)	17.0	(15.3–18.9)	65.3	(62.9–67.5)
**≥65 years**	0.4	(0.2–0.9)	31.4	(29.0–33.9)	48.5	(45.8–51.2)	19.7	(17.6–21.9)	68.2	(65.6–70.6)
Low education group	0.2	(0.0–1.5)	31.5	(21.3–43.8)	43.9	(32.6–55.8)	24.4	(15.5–36.4)	68.3	(56.0–78.5)
Medium education group	0.6	(0.2–1.5)	29.7	(26.3–33.2)	49.6	(45.9–53.4)	20.2	(17.4–23.3)	69.8	(66.2–73.2)
High education group	0.3	(0.1–1.0)	34.4	(32.1–36.7)	48.5	(46.0–51.0)	16.8	(15.0–18.8)	65.3	(63.0–67.6)

BMI=Body Mass Index, CI=confidence interval
